# A classification system for zebrafish adipose tissues

**DOI:** 10.1242/dmm.025759

**Published:** 2017-06-01

**Authors:** James E. N. Minchin, John F. Rawls

**Affiliations:** 1 Department of Molecular Genetics & Microbiology, Duke University, Durham, NC 27710, USA; 2 Department of Cell Biology & Physiology, University of North Carolina at Chapel Hill, Chapel Hill, NC 27599, USA; 3 British Heart Foundation Centre for Cardiovascular Science, University of Edinburgh, Edinburgh EH16 4TJ, UK

**Keywords:** Zebrafish, Adipose tissue, *In vivo* imaging, Classification system

## Abstract

The zebrafish model system offers significant utility for *in vivo* imaging of adipose tissue (AT) dynamics and for screening to identify chemical and genetic modifiers of adiposity. In particular, AT can be quantified accurately in live zebrafish using fluorescent lipophilic dyes. Although this methodology offers considerable promise, the comprehensive identification and classification of zebrafish ATs has not been performed. Here, we use fluorescent lipophilic dyes and *in vivo* imaging systematically to identify, classify and quantify the zebrafish AT pool. We identify 34 regionally distinct zebrafish ATs, including five visceral ATs and 22 subcutaneous ATs. For each of these ATs, we describe detailed morphological characteristics to aid their identification in future studies. Furthermore, we quantify the areas for each AT and construct regression models to allow prediction of expected AT size and variation across a range of developmental stages. Finally, we demonstrate the utility of this resource for identifying effects of strain variation and high-fat diet on AT growth. Altogether, this resource provides foundational information on the identity, dynamics and expected quantities of zebrafish ATs for use as a reference for future studies.

## Abstract (precis)

**Summary:** A standardized nomenclature and classification system for zebrafish adipose tissues and regression models to predict expected adipose size during the course of zebrafish development.

## INTRODUCTION

The chronic consumption of hypercaloric diets in modern societies causes excessive accumulation of lipid within adipose tissue (AT), AT dysfunction and increased risk for cardiovascular disease and diabetes (Klöting and Blüher, 2014; Guilherme et al., 2008). A central challenge in biomedical science is to understand how excessive AT expansion and ensuing dysfunction mediate susceptibility to cardiometabolic disease. Accumulation of AT around the abdomen, primarily within visceral AT (VAT; AT surrounding visceral organs) and abdominal subcutaneous AT (SAT; AT between abdominal muscle and skin), is positively associated with metabolic disease risk (Kahn et al., 2001; Karpe and Pinnick, 2015), whereas lower body gluteofemoral SAT is inversely associated with metabolic disease risk (Manolopoulos et al., 2010; Karpe and Pinnick, 2015). Accordingly, the ratio of upper to lower body AT, and also VAT to SAT, is a strong predictor of metabolic disease (Fox et al., 2007; McLaughlin et al., 2011; Goodpaster et al., 2005). In support, surgical reduction of VAT improves insulin sensitivity (Thorne et al., 2002; Csendes et al., 2009), whereas SAT removal exacerbates metabolic dysfunction (Foster et al., 2013). VAT promotes insulin resistance by exposing the liver to non-esterified fatty acids and pro-inflammatory factors via the hepatic portal vein (Item and Konrad, 2012; Rytka et al., 2011). Conversely, expansion of SAT protects ectopic lipid deposition in liver, skeletal muscle and pancreatic β-cells (Heilbronn et al., 2004; Porter et al., 2009). Understanding how regional ATs promote disease is likely to provide new avenues for therapeutic intervention.

AT is a vertebrate innovation and forms during development (Gesta et al., 2007). In both humans and mice, SAT is first detected in the embryo, before VAT develops postnatally (Poissonnet et al., 1984; Wang et al., 2013). Intriguingly, ATs derive from distinct developmental lineages (Hilton et al., 2015; Billon et al., 2007) and possess unique transcriptional signatures (Vohl et al., 2004; Gesta et al., 2006), suggesting that developmental mechanisms control regional AT deposition and function. Zebrafish offer a tractable model system to study AT development and regional deposition. Zebrafish AT is morphologically homologous to mammalian white AT (WAT) (Flynn et al., 2009; Song and Cone, 2007) and also expresses many markers indicative of mammalian WAT (Flynn et al., 2009; Imrie and Sadler, 2010; Oka et al., 2010). Furthermore, zebrafish AT responds to manipulation of energy balance, suggesting conserved functional homology to mammalian WAT (Minchin et al., 2015; Meguro et al., 2015; Leibold and Hammerschmidt, 2015; Flynn et al., 2009). Detailed classification of distinct AT depots in humans has helped facilitate study of regional AT localization and form a consistent nomenclature (Shen et al., 2003). VAT and SAT have been identified in zebrafish (McMenamin et al., 2013; Imrie and Sadler, 2010; Meguro et al., 2015; Minchin et al., 2015); however, a standardized classification system for zebrafish ATs has not yet been developed. The regional distribution of ATs can be visualized in live zebrafish using fluorescent lipophilic dyes (FLDs) (Minchin and Rawls, 2011), and the two-dimensional area of FLD-labelled AT is an accurate proxy for triacylglyceride (TAG) content (Tingaud-Sequeira et al., 2011). These studies suggest that FLD staining coupled with AT area quantification will provide a tractable system for studying zebrafish adiposity amenable to large-scale chemical and genetic studies.

In this resource article, we use FLDs and whole-animal *in vivo* imaging systematically to identify, classify and quantify zebrafish ATs. We identify 34 distinct ATs and classify them according to anatomical location. Furthermore, we quantify the area of each AT over a range of postembryonic zebrafish sizes and construct regression models to predict expected AT sizes. These data provide vital information on the expected complement and size of zebrafish ATs. Finally, we apply this classification and quantification system to identify effects of strain variation and diet on zebrafish AT. Altogether, the new approach established in this resource provides the framework for large-scale analysis of AT distribution dynamics and supports the expanded use of zebrafish as a model for AT research.

## RESULTS

### Characteristics of the wild-type cohort used to identify zebrafish adipose tissues

**Fig. 1.**
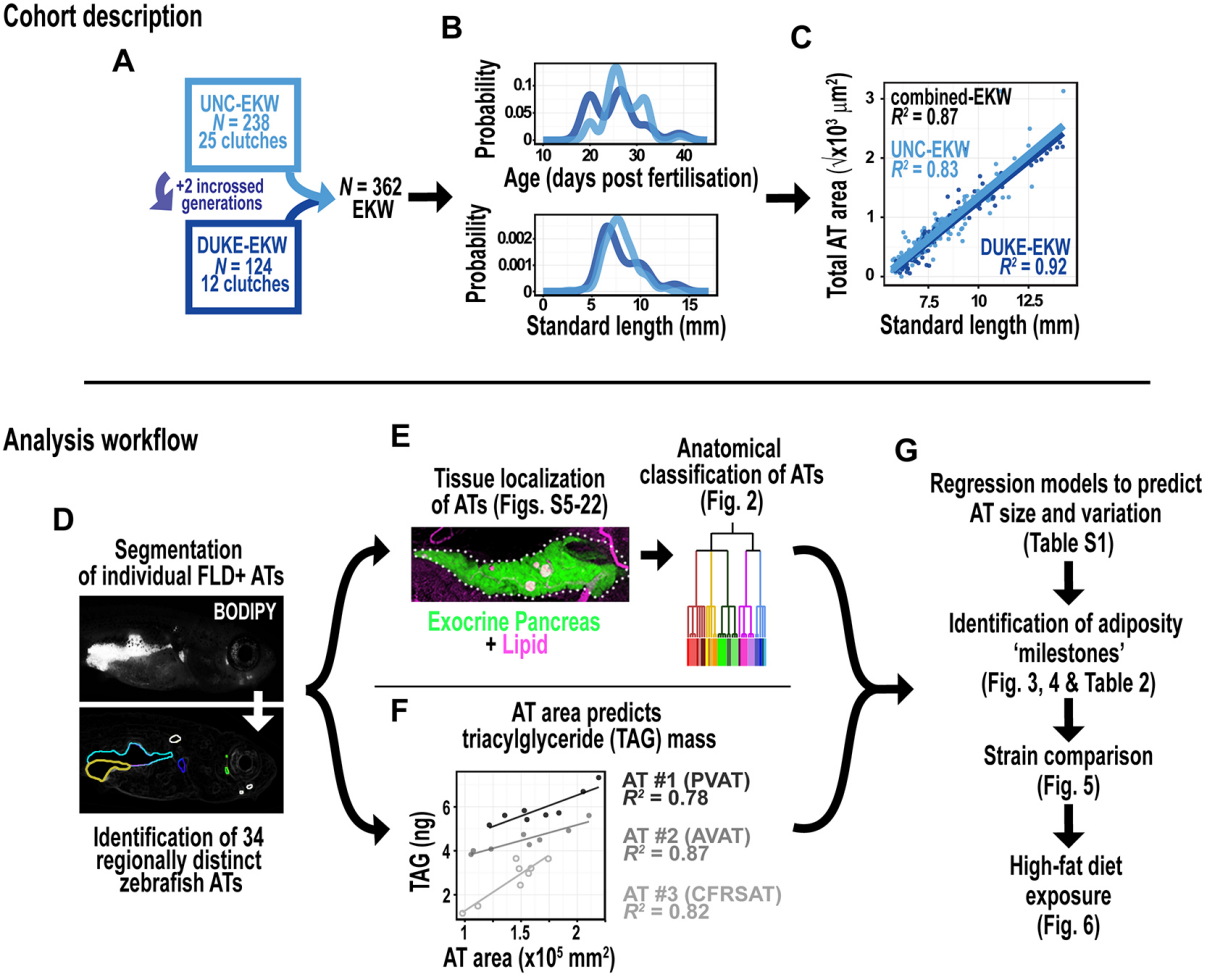
**Outline of animal cohorts and analysis workflow.** (A) Three hundred and sixty-two postembryonic EKW zebrafish from two institutions, University of North Carolina (UNC-EKW) and Duke University (Duke-EKW), were collected for this study. (B) Probability density functions illustrating the similar age (dpf) and SL of the UNC-EKW and Duke-EKW cohorts. (C) SL was an accurate predictor of total AT area (the sum of all AT areas) in both cohorts (combined, *n*=362, *R*^2^=0.87; UNC-EKW, *n*=238, *R*^2^=0.83; Duke-EKW, *n*=124, *R*^2^=0.91; all *P*<0.0001). Total AT was square root transformed to linearize the relationship. The relationship between SL and total AT between cohorts was not different by ANCOVA (*F*_1,358_=0.4855, *P*=0.486). (D-F) Each fish in the cohort was processed along a similar workflow. (D) First, each fish was stained with Nile Red or BODIPY, and regionally distinct ATs identified and segmented. (E) Each AT was then anatomically classified using information from histology or co-localization to transgenic reporter lines. An example is shown of LipidTOX staining of lipid in a transgenic line expressing GFP in the exocrine pancreas (*ptf1a:eGFP*). (F) We found that FLD^+^ area was an accurate predictor of lipid content for three regionally distinct ATs (*n*=8) (TAG=triacylglyceride). ATs were PVAT, AVAT and CFRSAT. (G) Therefore, for each AT we quantified FLD area and (1) constructed regression models to predict AT size at a given SL, (2) identified adiposity ‘milestones’ as indicators of stage, (3) compared adiposity traits in genetically distinct strains, and (4) evaluated the effect of a high-fat diet on adiposity traits.

We first established a large wild-type zebrafish cohort on which to conduct FLD staining and systematically to identify zebrafish ATs. We used 362 postembryonic Ekkwill (EKW) wild-type fish derived from 37 independent clutches (Fig. 1A). These EKW fish were aged between 20 and 39 days post-fertilization (dpf) and ranged between 4.2 and 14.2 mm standard length (SL). We chose to concentrate on postembryonic zebrafish, before overt sexual differentiation, as ATs initially appear during this period and expand to form a diverse array of ATs at distinct anatomical sites. The EKW fish were raised in two different locations: University of North Carolina (UNC-EKW) and Duke University (Duke-EKW). Duke-EKW were derived from the UNC-EKW stock and were separated by two incrossed generations (Fig. 1A). In addition to genotypic differences, rearing methods also differed between cohorts (see Materials and Methods for details). However, at the point of analysis, the cohorts were highly comparable with respect to age and size (Fig. 1B). Furthermore, the relationship between SL and total adiposity was not significantly different between cohorts (Fig. 1C; *P*=0.486), suggesting that these cohorts were comparable and could be analysed together. To help relate AT development to established postembryonic stages, we mapped EKW SL to standardized SL (SSL) (Table 1) and used the ‘composite staging’ convention previously proposed (Parichy et al., 2009).

**Table 1.**
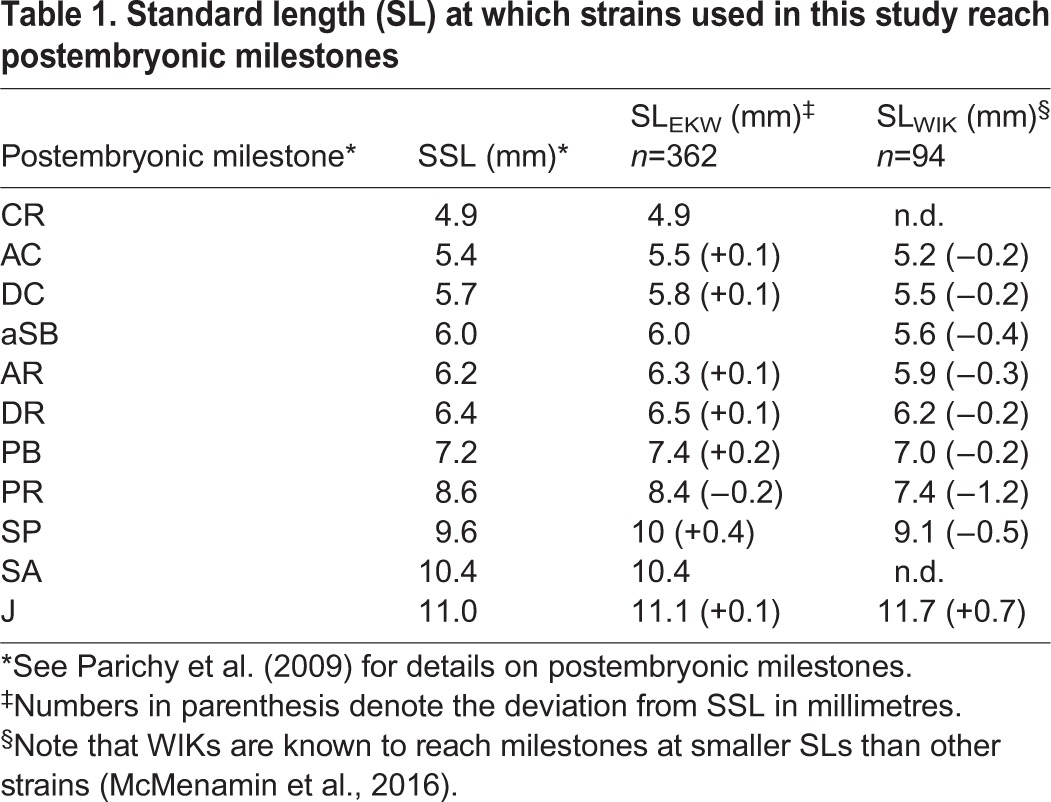
**Standard length (SL) at which strains used in this study reach postembryonic milestones**

### Identification and classification of zebrafish adipose tissues

**Fig. 2.**
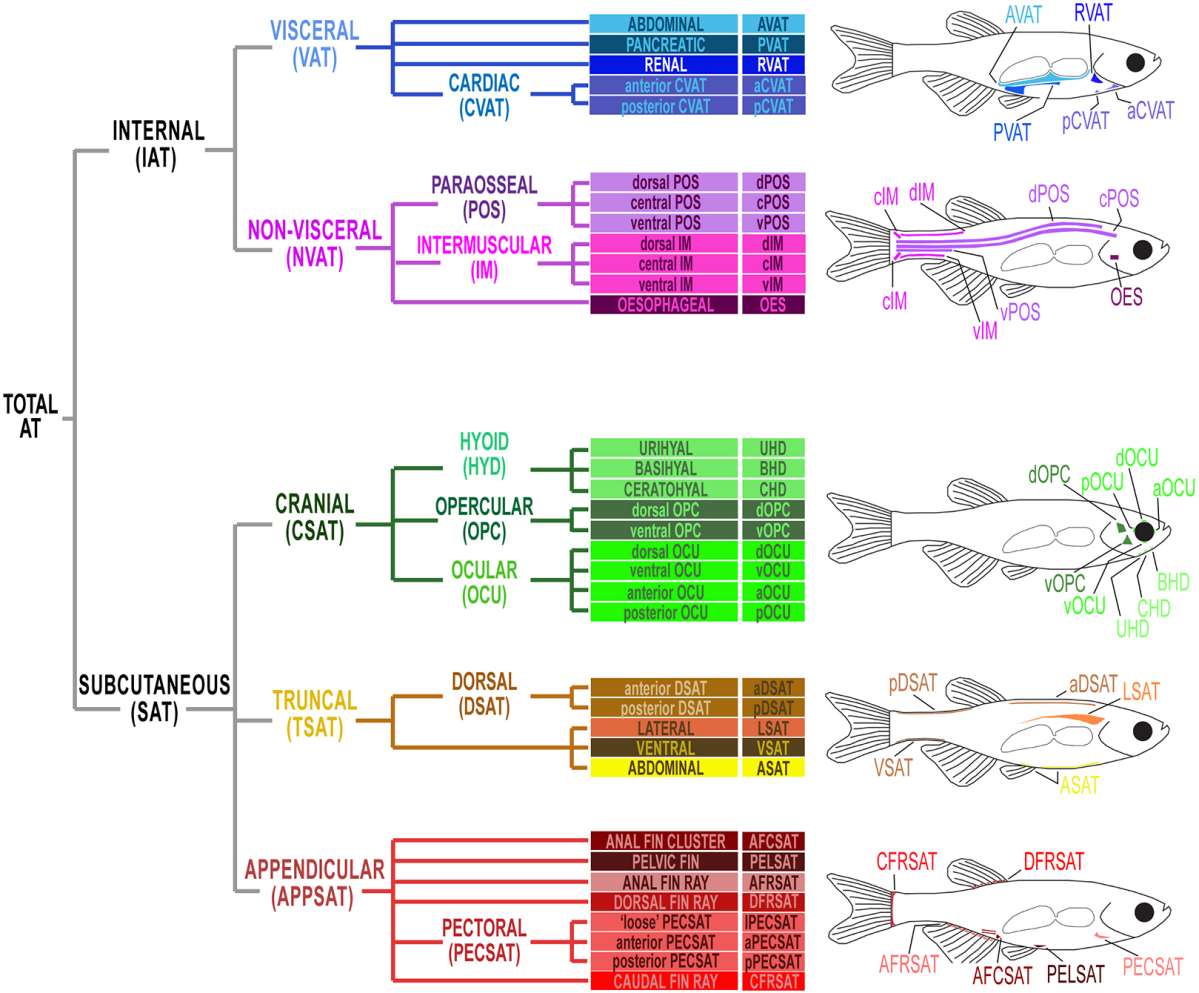
**Anatomical classification and location of zebrafish adipose tissues.** A schematic diagram illustrating the 34 regionally distinct zebrafish ATs identified by FLDs in this study. Drawings of zebrafish adapted from Minchin and Rawls (2017) with permission from Elsevier. These images are not published under the terms of the CC-BY licence of this article. For permission to re-use, please see Minchin and Rawls (2017).

Zebrafish possess many regionally distinct ATs; however, little is known regarding their anatomical location. Furthermore, a comprehensive AT classification system does not exist and standardized nomenclature has not been established. FLD staining of the 362 EKW fish within our cohort identified 34 regionally distinct zebrafish ATs (Fig, 1D; Fig. 2; Table 2). Using histology and localization to transgenic reporter lines, these ATs were classified according to anatomical location and relatedness (Fig. 1E, Fig. 2; Figs S5-S22). For classification purposes, we adapted a system previously used for human ATs (Shen et al., 2003). Accordingly, total zebrafish AT was first divided into two domains: internal AT (IAT), located internally, and subcutaneous AT (SAT), located between the dermis and the aponeuroses and fasciae of the muscles (Fig. 2; Table 2). Of the 34 ATs identified, 22 were classified as SAT and 12 as IAT (Fig. 2; Table 2). IAT was divided into visceral IAT (VAT; AT associated with internal visceral organs) and non-visceral IAT (NVAT; IAT−VAT=NVAT) (Fig. 2 and Table 2). SAT was found in many locations and grouped into three major divisions: truncal (TSAT; associated with the zebrafish trunk), cranial (CSAT; within the zebrafish head) and appendicular (APPSAT; associated with fins) (Fig. 2 and Table 2). An overview of regional AT location is given in Fig. 2, a summary of the classification system is provided in Table 2, and evidence for each of the anatomical classifications, along with descriptions on the morphology and growth of each AT is provided below and in Figs S5-S22.

**Table 2.**
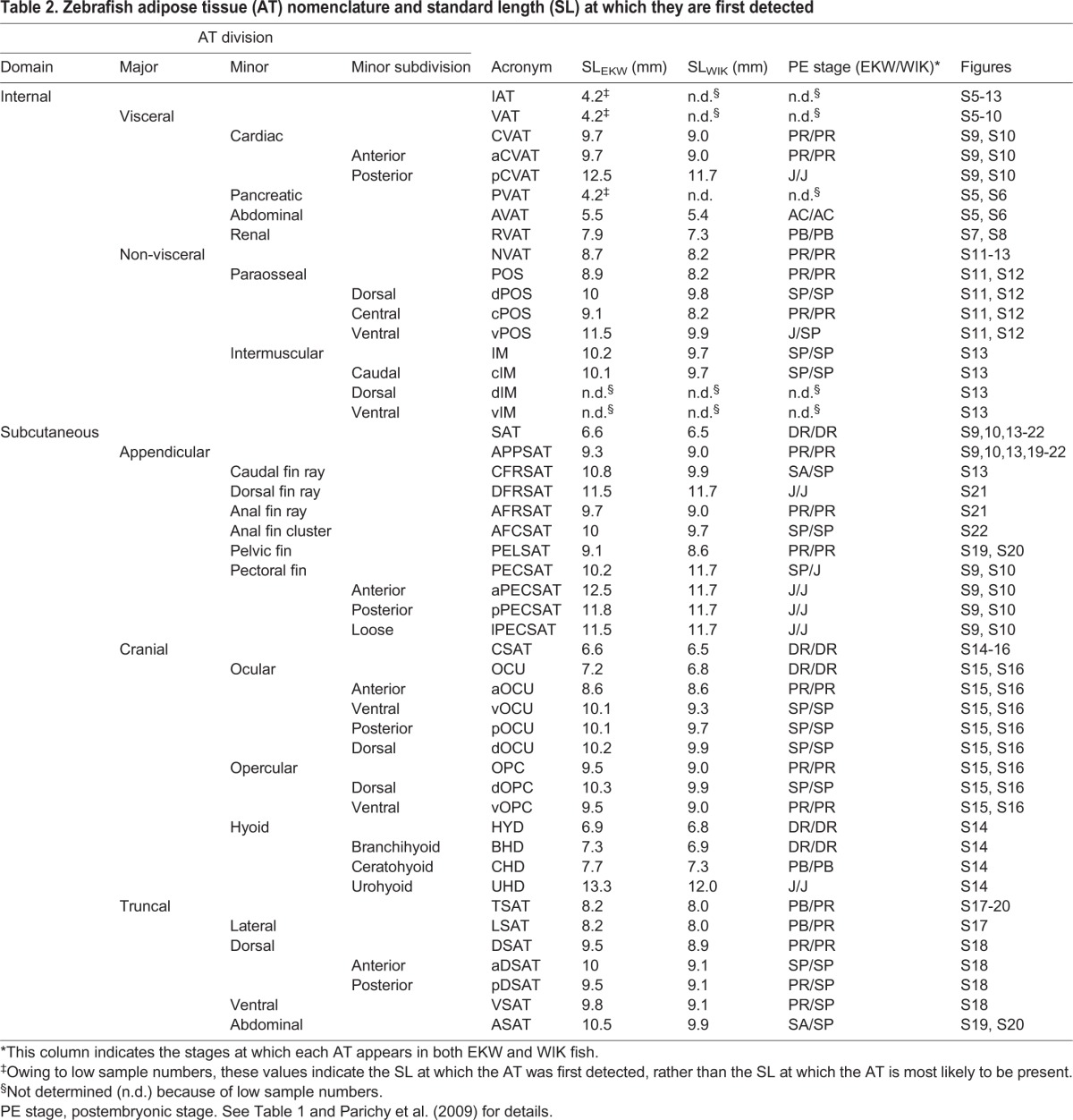
**Zebrafish adipose tissue (AT) nomenclature and standard length (SL) at which they are first** **detected**

### Area measurements accurately estimate the size of individual zebrafish adipose tissues

Previous studies have shown that measuring whole-animal FLD^+^ area accurately predicts whole-animal lipid mass (Tingaud-Sequeira et al., 2011). We reasoned that this method could be applied to individual ATs. As proof of principle, we measured the FLD^+^ area of three distinct ATs [pancreatic visceral adipose tissue (PVAT), abdominal visceral adipose tissue (AVAT) and caudal fin ray subcutaneous adipose tissue (CFRSAT)], then dissected each AT and quantified triacylglyceride content by fluorimetric assay (Fig. 1F). For each of the ATs, FLD^+^ area was an accurate predictor of triacylglyceride mass (*R*^2^=0.78, 0.87 and 0.82; all *P*<0.0001; Fig. 1F), thus supporting area measurements of individual ATs as an accurate estimator of lipid content.

### Regression analyses to predict normal size and variation of zebrafish adipose tissues

We next systematically quantified all identified zebrafish ATs within our EKW cohort. For each of the 34 ATs, we constructed regression models to predict AT size across a broad range of postembryonic EKW fish (Table S1). In addition, we also applied models to predict consolidated AT categories (i.e. VAT and SAT) (Table S1). SL explained the majority of variance for the 43 adiposity traits tested (98% of traits with *R*^2^>0.5; mean *R*^2^=0.84) (Table S1). Models for the single adiposity trait for which SL was not an accurate predictor (urohyoid, UHD, *R*^2^=0.14) could not be improved by use of different, or multiple, predictor variables [i.e. other measures of zebrafish size, including body area (BA) and height at the anterior margin of the anal fin (HAA); not shown]. Intriguingly, however, age had the greatest ability to predict UHD size (Table S3). For all ventrally located ATs, including UHD, other hyoid apparatus (HYD) ATs [basihyoid (BHD) and ceratohyal (CHD)] and subcutaneous abdominal adipose tissue (ASAT), SL had a reduced predictive ability (mean *R*^2^=0.51), suggesting that measurement of these depots from a lateral view was prone to experimental error (Table S1). However, overall these statistical models allow the prediction of expected AT levels in wild-type animals of different sizes. Charts depicting the growth dynamics for each AT are included in Figs S5-S22, and the raw measurement data are included in Table S4.

### General dynamics of adipose tissue formation and growth in zebrafish

**Fig. 3.**
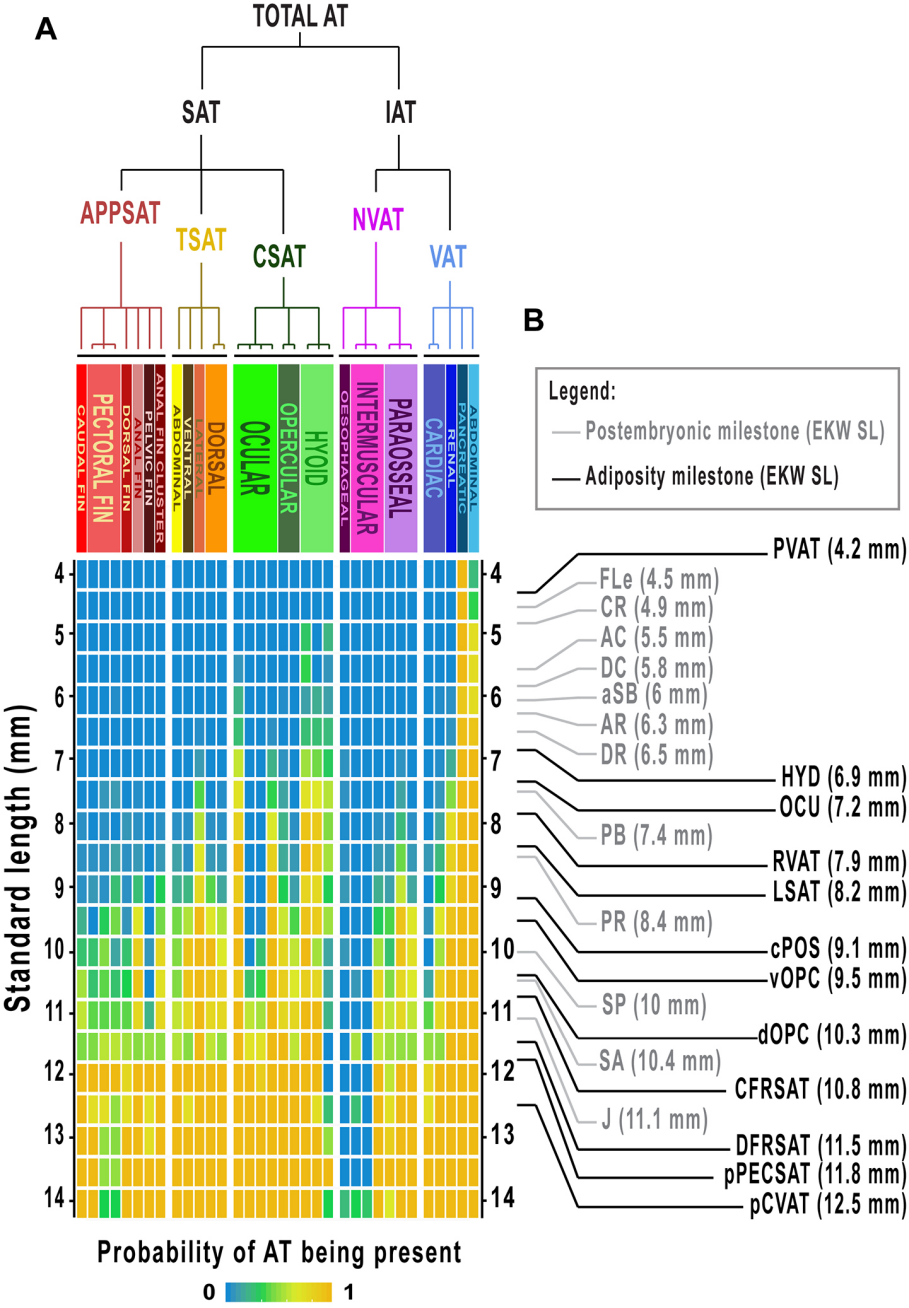
**Identification of adiposity ‘milestones’ as indicators of developmental progression.** (A) Heatmap indicating the likelihood of specific ATs being deposited relative to SL. The 362 fish were grouped into 0.5 mm bins according to SL. *n*=8-10 fish per bin. The tree is rotated 90° but otherwise identical to Fig. 2. (B) Integration of postembryonic milestones and the adiposity milestones identified in this study. The indicated SLs are for EKW fish, but can be converted for WIK fish using Tables 1 and 2.

**Fig. 4.**
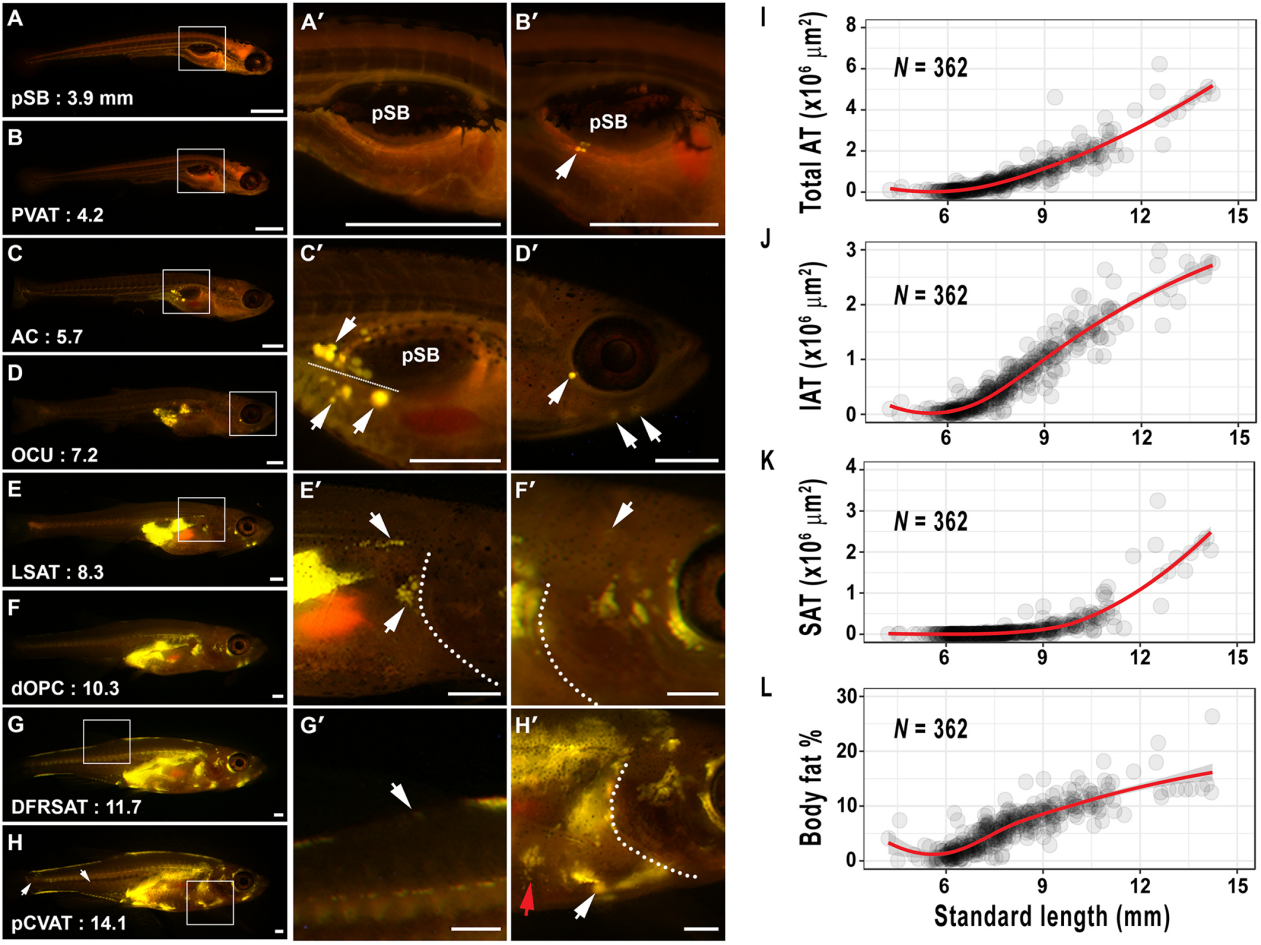
**Adipose tissue growth dynamics in postembryonic zebrafish.** (A-H′) Stereoscopic images of Nile Red-stained postembryonic zebrafish at stages indicated in Fig. 3B. The white boxes indicate the location of magnified images in the middle and right panels. The first AT to appear is PVAT (arrow in B′), followed by AVAT (upper arrow in C′; lower arrows beneath the dotted line indicate PVAT). CSAT is formed in the head (arrows indicate OCU or HYD ATs). (E′) LSAT (upper arrow) and RVAT (lower arrow) are detected on the zebrafish flank, and dotted line indicates the edge of the operculum. (F′) dOPC is deposited in dorsal cranial regions (arrow). (G′) DFRSAT is deposited at the dorsal fin (arrow). (H′) pCVAT is deposited on the ventral trunk in close proximity to the heart (white arrow; red arrow indicates lPECSAT). Dotted lines indicate the operculum boundary. (I) Total AT area relative to SL. (J) IAT area relative to SL. (K) SAT area relative to SL. (L) Body fat percentage relative to SL. Lines are fitted using locally weighted regression (LOESS) with 90% confidence regions shaded grey.

We next assessed the general dynamics of AT development in zebrafish. New ATs appeared throughout the EKW data set (Fig. 3A). Therefore, we reasoned that appearance of ATs could be used to define discrete ‘milestones’ relevant to stages of AT development. Using methodology defined by Parichy et al. (2009), we applied logistic regression to predict the SL and SSL at which each AT was most likely to appear in fish from our cohort (Fig. 3B; Table 2). The 26 resulting ‘milestones’ were evenly distributed throughout our data set (Fig. S1) and have utility for defining stages of zebrafish AT development. We interspersed adiposity ‘milestones’ with existing postembryonic ‘milestones’ to provide comprehensive coverage across postembryonic development (Fig. 3B). These discrete ‘stages’ are used throughout the remainder of the manuscript. Images of zebrafish representative for these adiposity milestones revealed the substantial increase in total AT and regional diversification (Fig. 4A-H′). Although total AT area steadily increased throughout the data set (Fig. 4I), growth within distinct AT domains revealed strikingly different trajectories. IAT was the first to be deposited at ∼PVAT:4.2 mm SL (milestone:SL) (Table 2) and underwent uninhibited growth until approximately renal visceral adipose tissue (∼RVAT):8 mm SL, at which point the rate of IAT accumulation began to slow (Fig. 4J). By contrast, SAT did not appear until dorsal fin ray appearance (DR) ∼DR:6.6 mm SL (Table 2) and underwent uninhibited expansion throughout the rest of the data set (Fig. 4K). These contrasting dynamics led to SAT gradually assuming a greater proportion of total AT, until IAT and SAT each contributed ∼50% of total AT in larger animals (Figs S2,S3). IAT was largely composed of VAT, with NVAT never contributing >12% of IAT (Fig. S3B). However, SAT composition was more dynamic and evenly distributed between the three main subdivisions (Fig. S3C). PVAT and AVAT were the primary components of IAT, whereas subcutaneous lateral adipose tissue (LSAT) was the largest SAT (Fig. S2B). Analysis of body fat percentage (total AT area as a percentage of body area) showed a marked slowing, coincident with the appearance of SAT (Fig. 4L), suggesting that in zebrafish, VAT is deposited first and rapidly expands before its growth slows coincident with the appearance and rapid diversification of SAT.

### Comparison of adiposity dynamics between zebrafish strains

**Fig. 5.**
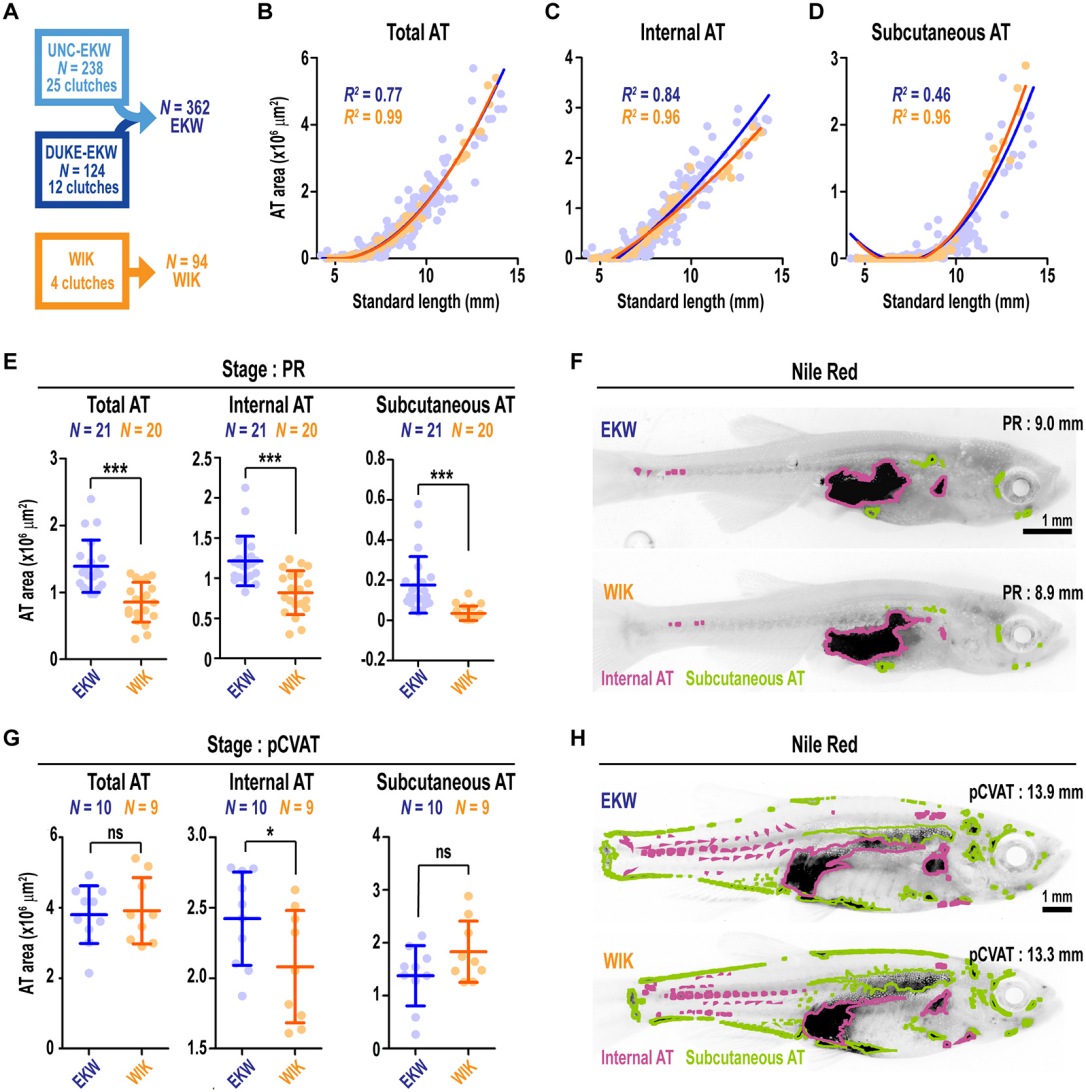
**Comparison of adiposity dynamics between EKW and WIK wild-type zebrafish strains.** (A) The 362 EKW zebrafish were compared with 94 WIK zebrafish derived from four independent clutches. (B-D) Scatterplots depicting total AT (B), internal AT (C) and subcutaneous AT (D) area relative to SL. Lines were fitted with polynomial regression. (E) Pairwise comparison of total AT, internal AT and subcutaneous AT between PR stage EKW and WIK zebrafish. (F) Representative images of Nile Red-stained PR stage zebrafish. Images are greyscale, inverted, and ATs are false coloured according to their classification. (G) Pairwise comparison of total AT, internal AT and subcutaneous AT between pCVAT stage EKW and WIK zebrafish. (H) Representative images of Nile Red-stained pCVAT stage zebrafish. Images are greyscale, inverted, and ATs are false coloured according to their classification. Groups were compared using Student's *t*-tests. *P<0.05; ***P<0.0001.

We next ascertained whether adiposity dynamics were comparable across different genetic backgrounds. To do so, we used the AT classification system described above and quantified ATs from 94 WIK wild-type zebrafish derived from four independent clutches (Fig. 5A). WIKs possessed all ATs present in EKWs, and vice versa (Table 2). Neither strain exhibited ‘strain-specific’ AT deposits (not shown). Furthermore, the configuration of ATs was identical between the strains (Fig. 5F,H). In accord with McMenamin et al. (2016), WIKs attained developmental stages at slightly smaller SL than either WT/WA or EKW strains (Table 1). However, the timing of AT appearance in WIKs was highly consistent with EKW (Table 2 and Table S2). Although not striking by eye, pelvic fin ray (PR) appearance stage (as per Parichy et al., 2009) WIK zebrafish (7.4–9.1 mm SL) had significantly reduced AT size when compared with equivalent stage-matched EKW fish (Fig. 5E,F). These reductions in adiposity were consistent across all AT categories (Fig. 5E,F). By contrast, at the stage of CVAT deposit at the posterior extent of the heart (pCVAT; >12.5 mm SL), WIKs had identical total AT when compared with stage-matched EKWs (Fig. 5G,H); however, IAT was reduced and SAT increased relative to EKWs (Fig. 5G,H). Taken together, the configuration and timing of AT development is essentially identical between EKW and WIK strains; however, WIKs display subtle differences from EKWs in AT size and distribution.

### Exposure to a high-fat dietary supplement preferentially increases ‘internal’ adiposity in pelvic fin bud stage postembryonic zebrafish

**Fig. 6.**
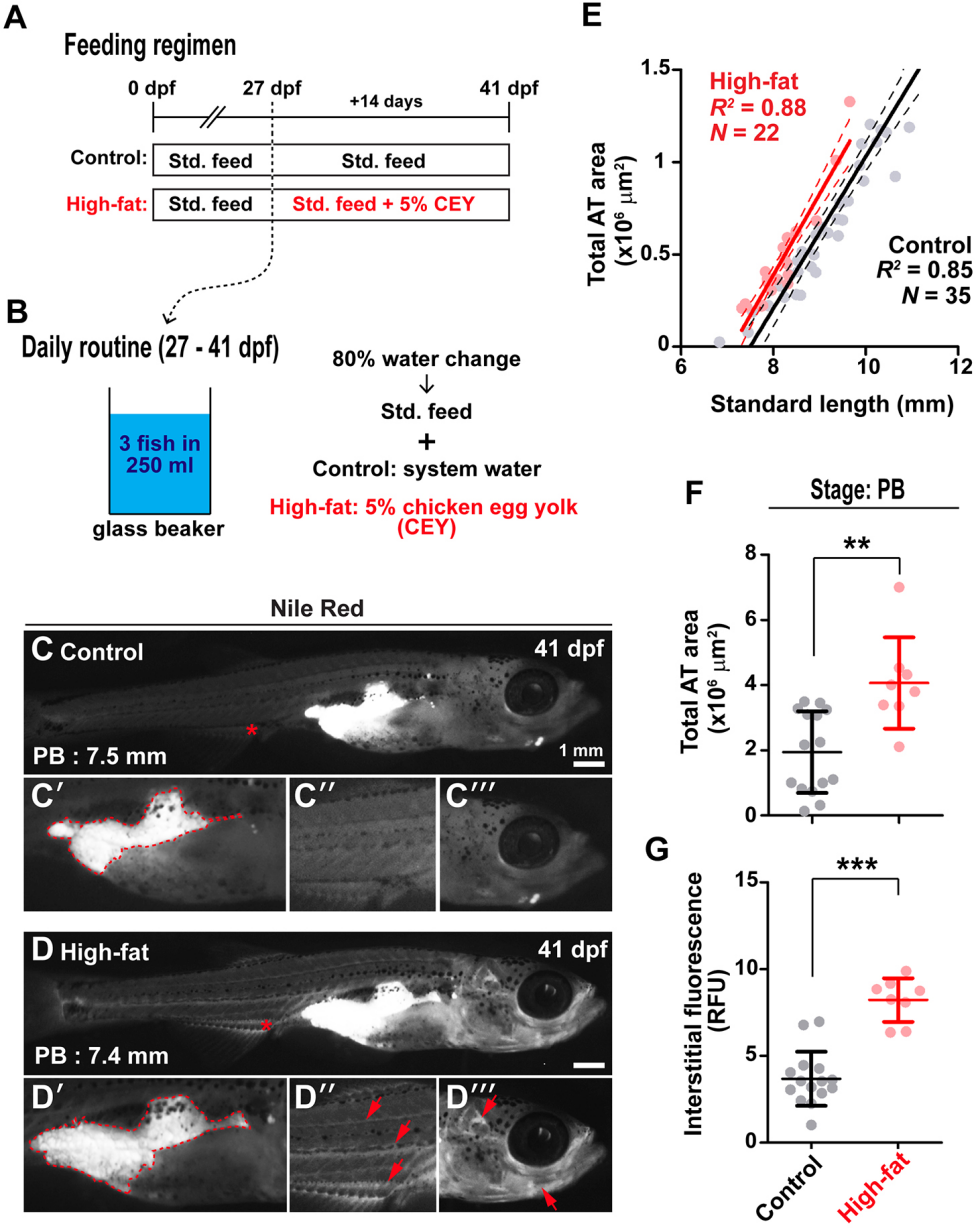
**Exposure to a high-fat diet preferentially increases internal AT in PB stage postembryonic zebrafish.** (A) Schematic diagram illustrating the experimental design. Groups were raised normally until 27 dpf, and then fed either a standard feed (Std. feed) or high-fat 5% chicken egg yolk (CEY). (B) From 27 dpf, fish were housed in glass beakers, with 80% daily water changes. (C-D‴) Representative images of 41 dpf fish fed either the Std. feed (control, C-C‴) or a high-fat diet (5% CEY, D-D‴). Arrows in C and D indicate the position at the anal fin that was quantified in G, and the position of images magnified in C″ and D″. The outlines in C′ and D′ indicate IAT (PVAT+AVAT), which was quantified in F. (E) Scatterplot indicating the increased total AT area in high-fat-fed fish (red) relative to control-fed fish (black). Lines are fitted using ordinary least squares (OLS) regression, with 95% confidence intervals. (F) Quantification of total AT area in control (black) and high-fat-fed fish (red). (G) Quantification of interstitial fluorescence. RFU=relative fluorescence unit and was normalized to background. Groups in F and G were compared using Student's *t*-tests. **P<0.001; ***P<0.0001. *n*=15 control PB stage fish, and *n*=8 high-fat-fed PB stage fish.

To evaluate the utility of the AT classification system further, we next sought to increase adiposity experimentally using 5% chicken egg yolk (CEY) as a high-fat diet supplement (Minchin et al., 2015; Semova et al., 2012; Carten et al., 2011). We exposed postembryonic zebrafish to 14 days of 5% CEY and applied the AT classification system to ascertain regional differences in AT response to a high-fat diet. From 27 dpf, fish were exposed to daily 2 h incubations in 5% CEY in addition to their standard feed (Std. feed) (Fig. 6A,B). After 14 days, fish exposed to the high-fat diet displayed slightly less somatic growth than control-fed animals (Fig. S4A); however, transitions between postembryonic stages and appearance of ATs were not significantly different between control and high-fat-fed fish (Fig. 6B,C). Furthermore, the body area of high-fat fish was identical to that of control-fed animals (Fig. 6D). High-fat diet-exposed animals had consistently larger total AT area when compared with control-fed animals (Fig. 6E,F). Furthermore, neutral lipid signal in interstitial regions was also increased after a high-fat diet (Fig. 6G), suggesting greater circulating lipid levels. Analysis of regional adiposity in PB stage fish revealed that VAT was preferentially enlarged relative to SAT (Fig. S4E,F), suggesting that regionally distinct ATs have different responses to high-fat diet.

### Evidence for the anatomical classification of zebrafish adipose tissues

To facilitate identification of zebrafish ATs, included below are detailed descriptions on the individual morphologies and growth patterns of each AT.

### Major division: internal visceral adipose tissues (VAT)

#### Minor division: pancreatic visceral adipose tissue (PVAT)

Pancreatic VAT (PVAT) was the first zebrafish AT to appear and was detected immediately ventral to the swim bladder (Fig. 4B,B′; Fig. S5A). We confirmed PVAT localization to the exocrine pancreas using the *ptf1a:eGFP* transgenic line (Fig. S5B-C′). The mechanism by which the pancreas promotes AT development is unclear; however, pancreatic acinar cells can transdifferentiate into adipocytes, suggesting the potential for a shared lineage (Bonal et al., 2009). Morphologically, PVAT initially appeared as a small cluster of lipid droplets (LDs) and underwent significant expansion and morphological change to assume a ‘saucepan-like’ morphology from squamation onset posterior (SP): ∼SP:10 mm SL (Fig. S5D-I′). This morphological change was consistent with similar changes occurring to the exocrine pancreas, and PVAT shape closely corresponded to exocrine pancreas shape (Fig. S5C,C′). It is likely that the morphological changes in PVAT and the exocrine pancreas occur simultaneously with gut looping, leading to the posterior pancreatic ‘bulb’ filling a gap created by the looped intestine. As PVAT was associated with the exocrine pancreas, it was asymmetrically localized to the right flank of larvae (Fig. S6A,B). However, from ∼SP:10 mm SL, small deposits of PVAT appeared on the left flank and exhibited distinct morphology consistent with a multi-lobed pancreas interspersed between a looped intestinal tract (Fig. S6A,B) (Chen et al., 2007). Owing to this asymmetry, PVAT was predominately located on the right flank, and analysis of only the left flank results in inaccurate quantification of PVAT and, consequently, also of VAT and IAT. In 4/362 EKW and 0/94 WIK fish, we observed switching of flank-specific PVAT morphology to the left flank, suggesting simple *situs inversus* occurs at a frequency of ∼1% in EKW stocks (Fig. S6A,B). The relationship between SL and PVAT area was nonlinear (Fig. S6F), and the rate of PVAT growth noticeably plateaued from ∼10 mm SL. Of note, AT was found immediately basal to the intestinal submucosa (Fig. S6E). In humans, omental AT juxtaposed to the intestine is often used for metabolic studies (Arner, 1995); however, histology suggested that AT proximal to the intestine was still associated with the exocrine pancreas and thus likely to be PVAT (Fig. S6E). In previous studies, PVAT was called pancreatic WAT (Imrie and Sadler, 2010; Tingaud-Sequeira et al., 2011), and adipocytes within PVAT were found to express markers of adipogenesis, including *peroxisome proliferator-activated receptor* γ (*pparγ*), *fatty acid-binding protein 11a* (*fabp11a*), *adiponectin* (*adipoqb*) and *adipsin* (*complement factor d*; *cfd*) (Flynn et al., 2009; Imrie and Sadler, 2010).

#### Minor division: abdominal visceral adipose tissue (AVAT)

Extensive intraabdominal VAT was also found in dorsal regions of the abdominal cavity associated with the swim bladders (SBs). We refer to this AT as abdominal VAT (AVAT) (Fig. S5A). AVAT was the second zebrafish AT to appear at ∼AC:5.5 mm SL and was often the largest AT (Fig. S5A; Fig. S6G). Although they become tightly pressed together in larger fish, a clear gap was visible between AVAT and PVAT until ∼10 mm SL when viewed laterally (Fig. S5F-H). In larger fish, when a gap was not evident, a distinguishing line dividing these ATs was often observed (Fig. S6C) (McMenamin et al., 2013). Analysis of AVAT on the left flank, where little PVAT is located, supported the view that these are distinct ATs (compare Fig. S5I with S6B). Taking these points into account, AVAT appeared entirely symmetrical which, together with its central location, might play a role in buoyancy and movements associated with swimming. AVAT first appeared at the posterior extent of the anterior SB and, subsequently, formed between the anterior and posterior SB lobes (Fig. S5D-I). Occasionally, a small AVAT cluster was located at the anterior end of the anterior SB, but as AVAT expanded, all three clusters merged to form a final AVAT with a consistent and distinctive morphology (compare Fig. S5D-I). Histology revealed that AVAT connected to the lateral extremes of the kidney (Fig. S6E,E″). Running bilaterally within the AVAT of adult males were testes, which themselves contain adipocytes; however, this distinct depot could not be discerned by whole-animal stereomicroscopy (Fig. S6E,E′). SL was a highly accurate predictor of AVAT area (Fig. S6G; Table 2). In previous studies, AVAT was called viscera WAT and found to express *pparγ*, *fabp11a*, *adipoqb* and *cfd* (Imrie and Sadler, 2010)*.*

#### Minor division: renal visceral adipose tissue (RVAT)

Renal VAT (RVAT) was a far smaller and simpler AT than either PVAT or AVAT. RVAT appeared immediately posterior to the operculum at ∼RVAT:7.9 mm SL (Fig. S7A and Table 2). RVAT retained a compact triangular shape until ∼SP:10 mm SL, at which point RVAT fractured into three or four distinct structures, perhaps as a result of increasing musculoskeletal complexity of the pectoral girdle (Fig. S7G,H). Histology revealed that RVAT could be located in an entirely subcutaneous location (Fig. S8C); however, at different anterior-posterior levels, RVAT expanded between body wall musculature (Fig. S8B) and was found contiguous with the kidney (Fig. S8A). Therefore, surprisingly, RVAT bridges both subcutaneous and visceral locations. In mammals, renal AT can be either perirenal (AT between the renal fascia and capsule) or pararenal (AT superficial to the renal fascia) (Lamacchia et al., 2011). By histology, zebrafish RVAT was directly opposed to the kidney, with no obvious membrane or connective tissue separating the two organs (Fig. S8A). Therefore, by morphology alone, our data suggest that RVAT is analogous to mammalian perirenal AT. As previously noted, a small portion of AVAT, at its dorsalmost extremity, was located in a perirenal position (Fig. S6E″). Similar to PVAT and AVAT, SL accurately predicted RVAT area (Fig. S7I; Table 2). In previous studies, RVAT adipocytes were shown to express *fabp11a* (Flynn et al., 2009).

#### Minor division: cardiac adipose tissue (CVAT)

In humans, cardiac VAT (CVAT) around the heart is found in two locations; epicardial (between the epicardium and the visceral layer of the pericardium) and pericardial (between the visceral and parietal pericardium) (Iacobellis, 2009). These different CVATs have distinct biomedical relevance (Iacobellis, 2009). In our data set, we observed AT only within the wall of the pericardium (Fig. S9); a location that is likely to correspond to pericardial AT in humans. Zebrafish CVAT was best viewed and quantified ventrally, and consisted of two deposits that grew to merge (Fig. S9A-D″). CVAT was first detected at the anterior heart (aCVAT) at approximately ventral opercular (∼vOPC):9.7 mm SL where the bilateral cleithrum meets ventrally (Fig. S9A-A″). A second CVAT deposit at the posterior extent of the heart (pCVAT) appeared from ∼pCVAT:12.5 mm SL (Fig. S9C-C″). aCVAT expanded posteriorly and connected with pCVAT to form a single depot with a distinct bulbous morphology when viewed ventrally (Fig. S9D′,D″). By histology, it was evident CVAT was contained within the wall of the parietal pericardium (Fig. S9G,H); however, even in adults the body wall musculature did not fully close around CVAT, suggesting that CVAT could also be classified as subcutaneous (Fig. S9G). CVAT was also in close proximity to ASAT (see below and Figs S19, S20). ASAT ran along the ventral midline from the anus to the heart (Fig. S19E) and was likely to join or closely abut CVAT. Unlike other VATs, the rate of CVAT growth did not decrease in larger fish, suggesting that CVAT undergoes uninhibited expansion throughout the fish from our cohort (Fig. S10).

### Major division: internal non-visceral adipose tissues (NVAT)

IAT is also classified as ‘non-visceral’ in humans (Shen et al., 2003). In zebrafish, when not associated with a visceral organ, we defined IAT as non-visceral (NVAT). NVAT primarily included AT associated with the skeleton (paraosseal, POS) and skeletal muscle (intermuscular, IM).

#### Minor division: paraosseal adipose tissue (POS)

Paraosseal AT (POS) was composed of multiple deposits associated with the zebrafish skeleton. When labelled by FLDs and viewed laterally, POS constituted three stripes that ran along the flank of zebrafish (Fig. S11A-G). We term these stripes according to their position along the dorsal-ventral axis; dorsal (dPOS), central (cPOS) and ventral (vPOS). cPOS was the largest POS and was initially detected in posterior locations at ∼cPOS:9.1 mm SL before spreading anteriorly (Fig. S11B-D). dPOS was detected at ∼SP:10 mm SL, again in a posterior location before extending anteriorly (Fig. S11E-G). vPOS was detected from approximately dorsal fin ray subcutaneous adipose tissue (∼DFRSAT):11.5 mm SL, and appeared as a small streak originating in posterior regions before terminating at the anal fin (Fig. S11G). All POS ATs appeared internal and segmented, suggesting that they are localized to reiterated structures (Fig. S11B-D,H-J; Fig. S12A). As such, cluster number and cluster length were useful metrics of POS development (Fig. S12B,C). By histology it was possible to discern that dPOS was located immediately dorsal to the neural tube (Fig. S11J-L) and was found in a large cluster at its anteriormost point, which we term the dPOS cluster (Fig. S11A,J). cPOS was localized around the outside of the notochord (Fig. S11K,L), and vPOS was present associated with ventral hemivertebrae (Fig. S11L). Quantification of FLD^+^ POS ATs appearance was variable (Fig. S12D-G), most probably because of experimental error attributable to its internal location. cPOS and dPOS were shown to express *fabp11a* by Flynn et al. (2009).

#### Minor division: intermuscular adipose tissue (IM)

AT associated with skeletal muscle can be in intramuscular (within a muscle), perimuscular (outside muscle) or intermuscular (between muscles) locations (Shen et al., 2003). Perhaps reflecting the limited resolution of our stereoscopic and histological methods, we only observed intermuscular (IM) AT in three locations within the zebrafish tail (Fig. S13). We term these IM ATs according to their position running dorso-ventrally at the midline of the tail; dorsal (dIM), central (cIM) and ventral (vIM) (Fig. S13A-D′). These deposits were usually very small and consisted of individual adipocytes when viewed on transverse section (Fig. S13E). *In situ* hybridization by Flynn et al. (2009) suggests that both dIM and vIM express *fabp11a*.

### Major division: cranial subcutaneous adipose tissue (CSAT)

CSAT was the first SAT to appear (at ∼DR:6.6 mm SL) and comprised a diverse and intricate collection of SATs that, for simplicity, we have subdivided into ATs located at the operculum (OPC), eye (ocular, OCU) and hyoid apparatus (HYD).

#### Minor division: hyoid (HYD)

Three distinct AT clusters were deposited in close proximity to the hyoid apparatus. These ATs were best viewed and quantified from ventral, and were classified according to their proximity to the closest hyoid structure: basihyoid (BHD), ceratohyal (CHD) and urohyoid (UHD) cartilages (Schilling et al., 1996) (Fig. S14). HYD ATs were named mandible WAT by Imrie and Sadler (2010). BHD and CHD area was readily observed and occurred in every animal larger than ∼7.3 and ∼7.7 mm SL, respectively (Fig. S14A-D′; Tables 2 and S4). However, the appearance of UHD was inconsistent and its area poorly estimated by SL and other measures of zebrafish size (Fig. S14I; Table 2 and not shown). When present, UHD signal was weak (compare Fig. S14C′,D′). Flynn et al. (2009) showed that BHD expresses *fabp11a*.

#### Minor division: opercular (OPC)

Two loose LD clusters were evident in dorsal and ventral regions of the operculum (Fig. S15). We term these dorsal opercular (dOPC) and ventral opercular (vOPC) ATs. Both dOPC and vOPC were fairly loose collections of adipocytes, but formed in very consistent anatomical positions (Fig. S15A-F′). SL accurately predicted OPC area (Fig. S16B). OPC were termed cranium WAT by Imrie and Sadler (2010).

#### Minor division: ocular (OCU)

Four distinct AT clusters formed around the eye from ∼PB:7.7 mm SL (Fig. S15 and Table 2), but these OCU clusters can be classified as a single structure for simplicity, and their appearance, growth and series of connections were highly consistent between fishes. Dorsal ocular AT (dOCU) initially consisted of two distinct clusters that connected to form an elongated AT across the dorsal eye (see #1 in Fig. S16C). Posterior ocular AT (pOCU) and ventral ocular AT (vOCU) were closely positioned (see #2 in Fig. S16C) and often merged in larger animals (Fig. S16C). Anterior ocular AT (aOCU) was sometimes observed in two clusters; immediately proximal to the eye and further dorsally (Fig. S15F,F′). OCU were termed cranium WAT by Imrie and Sadler (2010).

### Major division: truncal subcutaneous adipose tissue (TSAT)

#### Minor division: subcutaneous lateral adipose tissue (LSAT)

LSAT was located peripherally and ran laterally along the trunk at the horizontal myoseptum (Fig. S17). LSAT grew to become the largest zebrafish SAT (Fig. S17A-D″). LSAT first appeared at ∼LSAT:8.2 mm SL in an anterior location at the level of somite #1 (Fig. S17A,A″,F″). LSAT expansion occurred primarily along its anterior-posterior axis (Fig. S17A′-D′). However, expansion also occurred along its dorsal-ventral axis to produce a long, thin depot (Fig. S17D′). At the horizontal myoseptum, clusters of LSAT appeared constrained within somite boundaries (Fig. S17C″). By both histology and transgenic reporter methods, LSAT was partly localized to the wedge of slow-type skeletal muscle present at the horizontal myoseptum (Fig. S17E,F″). However, LSAT expanded ventrally and also juxtaposed fast-type fibres (Fig. S17E). SL was an accurate predictor of LSAT area (Fig. S17G); however, LSAT length (Fig. S17H,I) and width (Fig. S17J) could also be used to quantify LSAT development.

#### Minor division: subcutaneous dorsal adipose tissue (DSAT)

Extensive SAT was present in long, thin streaks in the dorsalmost trunk (DSAT) (Fig. S18). DSAT was categorized into anterior (aDSAT) and posterior (pDSAT) compartments intercepted by the dorsal fin (Fig. S18A-B′,C-F′). pDSAT was first detected at ∼vOPC:9.5 mm SL in a posterior location close to the caudal fin, and expanded anteriorly along its anterior-posterior axis (Fig. S18A-B′). aDSAT appeared at ∼SP:10 mm SL and initially consisted of two clusters; one located anteriorly towards the head (Fig. S18C′,D′) and another posteriorly at the base of the dorsal fin (Fig. S18C′,D′). These distinct aDSATs expanded and eventually connected to form a single aDSAT (not shown). Iridescent iridophores were especially prominent at aDSAT (Fig. S18E,F).

#### Minor division: subcutaneous ventral adipose tissue (VSAT)

In a similar manner to DSAT, SAT was also present in a ventral stripe, termed VSAT, between the anal and caudal fins (Fig. S18G-H′). VSAT appeared at ∼vOPC:9.8 mm SL (Fig. S18G′; Table 2) and expanded along its anterior-posterior axis until it connected the anal and caudal fins (Fig. S18G′). As with DSAT, VSAT was closely positioned with pigment cells; however, melanophores appeared most prominent around VSAT (Fig. S18H′).

#### Minor division: subcutaneous abdominal adipose tissue (ASAT)

From ∼SA:10.5 mm SL, SAT was located at the abdomen in a streak along the ventral belly (Figs S19, S20). We term this deposit ASAT. ASAT was initially located in a cluster close to the ventral region of PVAT (Fig. S19B-B″,E) and could be hard to distinguish from PVAT when viewed laterally (Fig. S19E). ASAT expanded within a restricted ventral region along the entire anterior-posterior extent of the zebrafish belly (Fig. S19B-E). ASAT did not fully fulfill the definition requirements of SAT, as it was not positioned between muscle and skin but, like CVAT, appeared as a fatty island surrounded by body wall musculature (Fig. S20A-C). ASAT connected with CVAT at its anterior extremity, suggesting that CVAT and ASAT are conjoining ATs and could potentially form a single large SAT (Fig. S19E,G). At the position of the pelvic fin girdle, ASAT narrowed and there appeared to be an imperfect ‘join’ between two ASAT parts (Fig. S19F). ASAT was called subcutaneous WAT by Imrie and Sadler (2010).

### Major division: appendicular subcutaneous adipose tissue (APPSAT)

Each zebrafish fin had associated lipid deposits located between or positioned at the base of fin rays.

#### Minor division: pelvic fin SAT (PELSAT)

Bilaterally symmetrical ATs appeared at the base of the pelvic fins at ∼cPOS:9.1 mm SL. We term these PELSAT (Figs S19, S20). PELSAT appeared as discrete clusters immediately anterior to the base of the pelvic girdle (Fig. S19A-A″) and grew to form symmetrical ‘wedges’ anterior to the pelvic girdle (Fig. S19B-D′). When viewed laterally, PELSAT could be mistaken for ASAT or even PVAT (Fig. S19E). In larger fish, ∼CFRSAT:11 mm SL, PELSAT formed loose adipocyte-LDs along its edges (Fig. S19D′). PELSAT was shown to express *fabp11a* by Flynn et al. (2009).

#### Minor division: anal fin ray SAT (AFRSAT)

Discrete LD clusters were evident at the base of anal fin rays, which we term anal fin ray SAT (AFRSAT) (Fig. S21). AFRSAT appeared at ∼vOPC:9.7 mm SL with only one or a few clusters initially evident (Fig. S21A-D′). In larger animals, the number of AFRSAT clusters increased, and clusters were evident between each fin ray (Fig. S21D). AFRSAT area was highly correlated with SL (Fig. S21G). Analysis of the number of clusters relative to SL revealed that additional AFRSAT clusters were added between fin rays during a short developmental time frame from ∼SP:10 mm SL (Fig. S21H).

#### Minor division: dorsal fin fay SAT (DFRSAT)

In a similar fashion to AFRSAT, adipocyte-LDs were clustered between the rays of the dorsal fin (Fig. S21I). Dorsal fin ray SAT, termed DFRSAT, appeared at ∼DFRSAT:11.5 mm SL and expanded by both adding new clusters and growth of existing clusters (Fig. S21I,J).

#### Minor division: anal fin cluster SAT (AFCSAT)

Positioned dorsally and anterior to AFRSAT was a complex collection of subcutaneous LDs that formed in a highly stereotypical fashion with consistent and distinctive morphology (Fig. S22). We term this collection of AT the anal fin cluster SAT (AFCSAT) (Fig. S22). AFCSAT appeared at ∼SP:10 mm SL and initially formed a large cluster at the anterior of the anal fin and immediately posterior to the anus (Fig. S22A-D,A″-D″). A second cluster appeared and extended into horizontal stripes in both anterior and posterior directions (Fig. S22A-D,A′-D′). Histology revealed that these horizontal stripes were in a subcutaneous location (Fig. S22F) and deposited at the base of the anal fin musculature (Fig. S22F). At later stages, these AFCSAT stripes also connected with sparse LDs forming ventral extensions of LSAT (Fig. S22D′).

#### Minor division: caudal fin ray SAT (CFRSAT)

AT at the base of the caudal fin (CFRSAT) appeared at ∼CFRSAT:10.8 mm SL (Fig. S13). Unlike AFRSAT and DFRSAT, CFRSAT was not deposited in discrete clusters between fin rays, but instead formed a continuous mass (Fig. S13A-D′). CFRSAT extended the full extent of the dorsal-ventral axis of the caudal fin, and connected to VSAT and pDSAT (Fig. S13D,D′). We defined the extent of CFRSAT at these extremes by its striated appearance relative to VSAT and pDSAT (Fig. S13D,D′,H). CFRSAT was shown to express *fabp11a* by Flynn et al. (2009).

#### Minor division: pectoral fin SAT (PECSAT)

The pectoral girdle supported three distinct AT clusters, which we termed anterior (aPECSAT), posterior (pPECSAT) and ‘loose’ (lPECSAT) (Fig. S9). When viewed laterally, aPECSAT and pPECSAT could be mistaken for CVAT (Fig. S9A-D). However, when viewed from ventral, aPECSAT was clearly observed close to aCVAT and positioned at the pectoral fin base from ∼pPECSAT:11.8 mm SL (Fig. S9A-A″). From ∼pCVAT:12.8 mm SL, pPECSAT was observed in more distal regions of the pectoral fin (Fig. S9C-D″). These PECSAT depots expanded but retained consistent morphology (Fig. S9D-D″). Loose LD clusters, collectively termed lPECSAT, were sometimes evident at more lateral regions of the pectoral fin from ∼DFRSAT:11.5 mm SL and formed large clusters (Fig. 4H′). SL was a poor predictor of PECSAT area, most probably because of the difficulties associated with measuring these depots laterally (Fig. S10D-G; Table 2).

## DISCUSSION

This resource establishes a new approach to the study of whole-animal adiposity dynamics in zebrafish, which we use as follows: (1) to identify 34 regionally distinct zebrafish ATs; (2) to describe detailed morphological characteristics for each of these ATs; (3) to identify ‘milestones’ useful for delineating postembryonic stages relevant to AT development in zebrafish; (4) to develop standardized nomenclature for zebrafish ATs; and (5) to construct statistical models that predict expected AT size and variation. Furthermore, we use this methodology to compare adiposity dynamics across zebrafish strains and analyse regional adiposity levels after exposure to a high-fat diet. Altogether, this study generates a reference resource useful for future studies investigating adiposity dynamics in zebrafish.

Of particular note, the EKW fish used in this study were derived from two highly related stocks, but fed different diets and raised in distinct facilities. Considering this environmental heterogeneity, it was surprising that variance in AT size was so small, suggesting that genotype exerts a strong influence on adiposity traits in zebrafish. This conclusion is supported by strong heritability estimates for human adiposity traits, including body fat distribution and early-onset obesity (O'Rahilly and Farooqi, 2008; Malis et al., 2005). Comparison of genetically distinct wild-type strains revealed essentially identical timing of the appearance and configuration of ATs. However, even though raised in identical environmental conditions, the size of ATs was different between the strains, further supporting a strong role for genotype in growth of zebrafish ATs. These data, coupled with (1) the ability to quantify a large range of adiposity traits in zebrafish, (2) the small unexplained variance in AT size when using the methods established in this article, and (3) the large sample sizes possible with zebrafish, suggest that quantitative studies investigating the genetic bases underlying adiposity traits are likely to be highly informative in zebrafish.

Previous studies have established that ATs in zebrafish and mammals share many homologous molecular markers (Flynn et al., 2009; Imrie and Sadler, 2010; Minchin et al., 2015). However, the classifications presented here are based purely on morphological characteristics. As a result, the functional and metabolic homologies between the distinct zebrafish and mammalian ATs remain unclear and could be addressed through transcriptomic and functional analyses. Indeed, recent work suggests that at least some of the molecular mechanisms governing regional AT distribution and physiology are conserved in zebrafish (Minchin et al., 2015; Ouadah-Boussouf and Babin, 2016; Leow et al., 2016). Intriguingly, mammalian AT is first formed in subcutaneous locations before VAT appears at later stages. However, in zebrafish, the converse is true. Although this difference might not be significant, it is interesting to speculate that VAT might fulfil advantageous roles during earlier stages of zebrafish postembryonic development. Examples of such roles could include supporting reproductive development (Leibold and Hammerschmidt, 2015) or growth and function of visceral organs. Altogether, the use of FLDs and stereomicroscopy retains amenability for large-scale studies and will provide a useful ‘first-pass’ assessment before more in-depth analysis of AT cellularity and function.

## MATERIALS AND METHODS

### Zebrafish strains and husbandry

All zebrafish experiments conformed to the US Public Health Service Policy on Humane Care and Use of Laboratory Animals, using protocols approved by the Institutional Animal Care and Use Committee of University of North Carolina-Chapel Hill and Duke University. Wild-type Ekkwill (EKW) strain zebrafish were used in this study. EKW fish were raised at either University of North Carolina-Chapel Hill (UNC-EKW) or Duke University (Duke-EKW) under a 14 h light/10 h dark cycle. WIK fish were raised at UNC.

UNC-EKW or WIK embryos were collected from adult pairs and ∼50 embryos raised in 100-mm-diameter Petri dishes containing 30 ml of system water at 28.5°C. At 5 dpf, embryos were transferred to static 2 litre tanks at a density of 10 larvae/l. At 10 dpf, tanks were moved onto a slow-drip water supply, and at 14 dpf the rate of water supply was increased to a steady flow. From 5 dpf, UNC-EKW larvae were fed twice daily a 3:2 powdered mixture of Active Spheres Golden Pearls (Brine Shrimp Direct, Ogden, UT) and spirulina powder (Aquatic Eco-Systems, Apopka, FL). At 10 dpf, the diet was changed to a 3:2 powdered mixture of Rotifer Size I Golden Pearls and spirulina-fed twice daily. From 14 dpf, powdered diets were supplemented with live *Artemia franciscana* (hereafter called *Artemia*; Aquafauna Bio-Marine), and larvae were gradually weaned onto an *Artemia*-only diet over the next few days. From 30 dpf, the *Artemia* diet was supplemented with a 5:2:1:1:1 mixture of TetraMin flakes, AquaTox flakes, spirulina, freeze-dried *Artemia* (Aquatic Eco-Systems) and Cyclopeeze (Argent Labs).

Duke-EKW embryos were collected from adult pairs and ∼50 embryos raised in Petri dishes containing 50 ml of embryo medium at 28.5°C. At 5 dpf, larvae were transferred to 3 litre tanks (10 larvae/l) with slow dripping water at a density of 20 larvae/l. From 5 to 14 dpf, larvae were fed Zeigler Larval AP100 (Zeigler Bros. Inc.) twice daily, supplemented with Gemma Micro 75 (Skretting) powder twice daily. From ∼14 dpf, larvae were fed *Artemia* twice daily supplemented with a twice daily feed of Gemma Micro 75. From 28 dpf, the Micro 75 diet was replaced with Micro 150. Postembryonic zebrafish were exposed to 5% chicken egg yolk (CEY) as described by Minchin et al., (2015). *Tg*(*ptf1a:eGFP*)*^jh1Tg^* and *Tg*(*smyhc1:eGFP*)*^i104^* transgenic strains (referred to as *ptf1a:eGFP* and *smyhc1:eGFP*) were maintained on EKW backgrounds (Elworthy et al., 2008; Pisharath et al., 2007). The ‘composite staging’ convention is used throughout this manuscript (Parichy et al., 2009). The stages used are documented in Fig. 3B and reported using the recommended ‘milestone:SL’ format. Conversion of EKW and WIK SL to SSL can be performed using Table 1.

### FLDs, image acquisition and analysis

BODIPY 505/515 (referred to as BODIPY; Life Technologies), Nile Red (Sigma Aldrich) and HCS LipidTOX Red (referred to as LipidTOX; Life Technologies) were used to label neutral lipid. LipidTOX dyes were preferred for both confocal analysis and when using fluorescent transgenic lines, as fluorescence emission from LipidTOX is restricted to single channels. BODIPY and Nile Red are brighter dyes and were preferred when using stereomicroscopy to image whole-animal adiposity. Staining was conducted as described by Minchin and Rawls (2011). Whole-animal FLD imaging followed Minchin and Rawls (2011) except that an eGFP bandpass filter was used to image both BODIPY and Nile Red dyes (Leica Microsystems). Confocal imaging followed Minchin et al. (2015).

All image analysis was conducted in FIJI/ImageJ (v1.50e) (Schindelin et al., 2012). Two copies of each image were opened (one for thresholding and one as an unmanipulated control image for comparison). The image scale was set, and image brightness and contrast were manually adjusted if necessary. SL and height at the anterior margin of the anal fin (HAA) were measured using the line tool according to Parichy et al. (2009). Body area (BA) was manually traced using the polygon tool (fins excluded). Image thresholding based on pixel intensity was performed to delineate AT area. For ATs that did not touch, the threshold was manually set using the slider until the area approximated the lipid dye. The magic wand tool was then used to select AT area and measured. For ATs that were touching, the polygon tool was used to trace the outline of each AT and area measured. For regions where the dividing line between depots was not visible, a straight line between the two farthest distinguishing points was drawn. Lateral view images were used for all measurements. Image acquisition settings were sufficient to detect FLD fluorescence in ATs, whilst excluding autofluorescence (Fig. S23).

### AT dissections and triacylglyceride quantification

The area of three ATs (PVAT, AVAT and CFRSAT) were measured in 24 zebrafish following the protocol above. The zebrafish were then individually housed in glass beakers overnight to allow the Nile Red stain to wash out. The following morning, ATs were dissected and triacylglyceride quantified using the fluorimetric triacylglyceride assay kit (Biovision) and a Synergy HT plate reader (BioTek).

### Histology

Fixation, embedding, sectioning and Masson's trichrome staining of juvenile and adult EKW zebrafish was conducted as described by Minchin et al. (2015). Juveniles were ∼14 mm SL and adults were ∼6 months of age. Alizarin Red staining was conducted as described by Valasek et al. (2011).

### Statistics

To determine the SL at which specific ATs appear, we performed logistic regression as per Parichy et al. (2009). A value of *P*>0.5 was used to determine when an AT was most likely to be present. Linear ordinary least squares (OLS) regression was used to test the ability of SL to predict AT area. To select the most accurate model, transformations to linearize nonlinear relationships and addition of power functions were tested. Full and reduced models were compared with a partial *F* statistic. Where error variances were unequal, observations were weighted with the absolute value of each residual. The best model for each AT is included in Table S1 (EKW) and S2 (WIK). As the models contain power functions, care must be taken in extrapolating beyond the range of the data or at data extremes.
